# Over-Expression of Hypochlorite Inducible Major Facilitator Superfamily (MFS) Pumps Reduces Antimicrobial Drug Susceptibility by Increasing the Production of MexXY Mediated by ArmZ in *Pseudomonas aeruginosa*

**DOI:** 10.3389/fmicb.2020.592153

**Published:** 2021-01-12

**Authors:** Punyawee Dulyayangkul, Naphat Satapoomin, Matthew B. Avison, Nisanart Charoenlap, Paiboon Vattanaviboon, Skorn Mongkolsuk

**Affiliations:** ^1^Program in Applied Biological Sciences: Environmental Health, Chulabhorn Graduate Institute, Chulabhorn Royal Academy, Bangkok, Thailand; ^2^School of Cellular and Molecular Medicine, University of Bristol, Bristol, United Kingdom; ^3^Laboratory of Biotechnology, Chulabhorn Research Institute, Bangkok, Thailand

**Keywords:** *Pseudomonas aeruginosa*, MFS, efflux pump, paraquat, ethidium bromide, MexXY, ArmZ

## Abstract

*Pseudomonas aeruginosa*, a well**-**known cause of nosocomial infection, is frequently antibiotic resistant and this complicates treatment. Links between oxidative stress responses inducing antibiotic resistance through over**-**production of RND**-**type efflux pumps have been reported in *P. aeruginosa*, but this has not previously been associated with MFS**-**type efflux pumps. Two MFS efflux pumps encoded by *mfs1* and *mfs2* were selected for study because they were found to be sodium hypochlorite (NaOCl) inducible. Antibiotic susceptibility testing was used to define the importance of these MFS pumps in antibiotic resistance and proteomics was used to characterize the resistance mechanisms involved. The results revealed that *mfs1* is NaOCl inducible whereas *mfs2* is NaOCl, N**-**Ethylmaleimide and t**-**butyl hydroperoxide inducible. Deletion of *mfs1* or *mfs2* did not affect antibiotic or paraquat susceptibility. However, over**-**production of Mfs1 and Mfs2 reduced susceptibility to aminoglycosides, quinolones, and paraquat. Proteomics, gene expression analysis and targeted mutagenesis showed that over**-**production of the MexXY RND**-**type efflux pump in a manner dependent upon *armZ*, but not *amgRS*, is the cause of reduced antibiotic susceptibility upon over**-**production of Mfs1 and Mfs2. *mexXY operon* expression analysis in strains carrying various lengths of *mfs1* and *mfs2* revealed that at least three transmembrane domains are necessary for *mexXY* over**-**expression and decreased antibiotic susceptibility. Over-expression of the MFS-type efflux pump gene *tetA*(C) did not give the same effect. Changes in paraquat susceptibility were independent of *mexXY* and *armZ* suggesting that it is a substrate of Mfs1 and Mfs2. Altogether, this is the first evidence of cascade effects where the over**-**production of an MFS pump causes over**-**production of an RND pump, in this case MexXY via increased *armZ* expression.

## Introduction

*Pseudomonas aeruginosa* is Gram-negative opportunistic human pathogen causing nosocomial infections. *P. aeruginosa* is found ubiquitously and is able to tolerate harsh environments (Stover et al., 2000). It is tolerate to stresses and antibiotics because it is well-equipped with intrinsic and acquired antibiotic resistance mechanisms, particularly drug efflux pumps (Poole, 2011; Morita et al., 2012). Based on the *P. aeruginosa* PAO1 genome sequence, Resistance-Nodulation-Division (RND) family and Major Facilitator Superfamily (MFS) efflux pumps are the two most abundant efflux systems (Stover et al., 2000). Both use proton gradients to extrude substrates (Piddock, 2006). RND pumps, commonly found in Gram-negative bacteria (Poole, 2008), are composed of three components spanning the inner and outer membranes (Piddock, 2006). The components are the inner membrane pump protein which is specific to particular substrates; an outer membrane protein and a periplasmic accessory protein that holds the inner and outer membrane proteins together and forms a channel allowing extrusion of the substrates outside of the cell (Piddock, 2006; Fernández and Hancock, 2012).

Among 12 annotated RND pumps in *P. aeruginosa*, the most clinically important antibiotic efflux pumps are MexAB-OprM, MexXY-OprM, MexCD-OprJ, and MexEF-OprN (Llanes et al., 2004; Lister et al., 2009; Morita et al., 2012; Rampioni et al., 2017; Colclough et al., 2020). MFS efflux pumps, which are found in both Gram**-**negative and Gram**-**positive bacteria are poorly studied in *P. aeruginosa.* This family of pumps includes those able to extrude a wide range of substrates including antibiotics and xenobiotics (Srijaruskul et al., 2015; Vattanaviboon et al., 2018). TetA, an MFS pump which is responsible for tetracycline resistance, is one of the most common antibiotic resistance mechanisms known, being encoded as a mobile gene that is common in many Enterobacteriaceae (Reisz et al., 2013; Grossman, 2016). There are many reports that stress can alter antibiotic susceptibility in various bacteria including *Escherichia coli*, *P. aeruginosa*, *Salmonella enterica* serovar Typhimurium, *Bacillus subtilis*, *Staphylococcus aureus*, *Mycobacterium tuberculosis, Klebsiella pneumoniae*, and etc. (Poole, 2012). In household bleach, NaOCl is used as a disinfectant. Furthermore, during host-microbe interaction, the host immune system, i.e., macrophages and neutrophils produce and release mixture of reactive oxygen species (ROS) including hypochlorous acid (HOCl) (Gray et al., 2013). It is therefore of particular interest to investigate whether oxidative stress can activate antibiotic efflux, because the effect may be to reduce antibiotic susceptibility at the site of infection. Based on our secondary analysis of published microarray data, two MFS efflux pumps, encoded by *mfs1* and *mfs2* were selected for study based on their observed induction in *P. aeruginosa* treated with NaOCl (Small et al., 2007). Herein, relationships between hypochlorite-inducible MFS efflux pumps and changes in antibiotic susceptibility in *P. aeruginosa* were examined. In so doing, we have uncovered a novel cascade effect, whereby MFS pump over**-**production leads to RND efflux pump over**-**production and reduced antibiotic susceptibility.

## Materials and Methods

### Bacterial Strains, Plasmids, Primers, and Growth Conditions

*E. coli* strains were cultivated at 37°C with 180 rpm agitation in Luria Bertani Broth (LB) (BD Difco, United States) supplemented with antibiotics if necessary whereas *P. aeruginosa* were cultivated in Mueller Hinton Broth (MHB) (BD Difco, United States). All strains, plasmids and primers used in this study are listed in Supplementary Table 1.

### Mutants, Complementation, and Over-Expression Strains

The *mexX* and *armZ* mutants were constructed by gene inactivation mediated by the pKNOCK suicide plasmid (Alexeyev, 1999). The *mexX* and *armZ* DNA fragments were amplified with Phusion High-Fidelity DNA Polymerase (NEB, United Kingdom) from *P. aeruginosa* PAO1 genomic DNA by specific primers. BT6223 (5′-GAGTACACCGAAGCGCAGAC-3′) and BT6224 (5′-GGCTGGGAGAAGTTCACGTA-3′) were used to amplify a *mexX* DNA fragment. BT7158 (5′-GACAAACTGGAAAAGCGCCT-3′) and BT7159 (5′- CCGTCTCGACCTGCTGTAG-3′) were used to amplify an *armZ* DNA fragment. Each PCR product was ligated into the pKNOCK-GM at the *Sma*I site. The recombinant plasmid in *E. coli* BW20767 (donor) was then transferred into wild-type *P. aeruginosa* PAO1 (recipient) cells by conjugation (Alexeyev, 1999). The transconjugant was selected using gentamicin (30 μg/ml) and the mutation was confirmed by Southern blotting (Sambrook and Russell, 2001).

The *amgRS* DNA fragment was amplified with Phusion High-Fidelity DNA Polymerase (NEB, United Kingdom) from *P. aeruginosa* PAO1 genomic DNA by specific primers BT7317 with a *Sac*I site included, underlined (5′-GGAGAGCTCTTCGGCGATAC-3′) and BT7318 (5′-AAGACCGCGGTGCTCGAGGGAAA-3′), digested with *Sac*I and ligated to pUC18 between the *Sac*I and *Hin*cII sites, generating pUC18:*amgRS*. A fraction of *amgRS* on pUC18:*amgRS* was excised using *Sal*I plus *Kpn*I and replaced with *lox*P-GM^*R*^-*lox*P excised from pCM351 (Marx and Lidstrom, 2002); resulting in pUC18GM containing flanking regions of *amgRS.*

A modified pUC18GM plasmid constructed in Laboratory of Biotechnology, Chulabhorn Research Institute (Somprasong et al., 2012), where *lox*P-GM^*R*^-*lox*P was excised from pCM351 (Marx and Lidstrom, 2002) by digestion with *Sac*I and *Eco*RI and inserted to pUC18 at the same sites was used to inactivate *mfs1* (*PA1262*) and *mfs2* (*PA1282*). First, sequences flanking each gene were generated by PCR. DNA fragments were amplified using Phusion High-Fidelity DNA Polymerase (NEB, United Kingdom) from *P. aeruginosa* PAO1 genomic DNA by gene specific primers of *mfs1* (BT6022-5) and *mfs2* (BT6026-9), listed in Supplementary Table 1. The two flanking regions for each gene were inserted into pUC18GM MCS, where *lox*P-GM^*R*^-*lox*P lies between the two flanking sequences. pUC18GM containing flanking regions of *mfs1, mfs2* or *amgRS* was electroporated into PAO1 sucrose competent cells (Choi et al., 2006). A double cross-over between the gene on the chromosome and the flanking regions on the plasmid results in a replacement of the gene with gentamicin resistant gene flanked by *lox*P sites. A plasmid carrying *cre* recombinase, pCM157 (Marx and Lidstrom, 2002), was then electroporated into the putative mutants to excise the GM^*R*^ cassette via the flanking *lox*P sites. Curing of pCM157 from the putative mutants was achieved through serial subcultures. The deletion mutants were confirmed by PCR.

Plasmid mediated complemented and over-expression strains were constructed by electroporation of pBBR1MCS-4 (Kovach et al., 1995) and pBBR1MCS-4 containing *mfs1* (pMfs1) or *mfs2* (pMfs2). In brief, *mfs1* and *mfs2* full length gene were amplified with Phusion High-Fidelity DNA Polymerase (NEB, United Kingdom) from *P. aeruginosa* PAO1 genomic DNA by specific primers listed in Supplementary Table 1. The PCR product was ligated into the pBBR1MCS-4 at the *Sma*I site. Each recombinant plasmid was then transferred into wild-type *P. aeruginosa* cells by electroporation. The complemented and over-expression strains were selected for carbenicillin resistance (200 μg/ml) and the mutation was confirmed by PCR using plasmid universal primers.

### Determination of Antibiotic Susceptibility Using Disk Diffusion

Disk diffusion assays were performed according to the Kirby-Bauer method with some modifications. Strains were grown overnight in MHB with selection as required and sub-cultured to MHB with an initial OD_600__*nm*_ of 0.1. Then, 50 μl of the log phase cells (OD_600__*nm*_ 0.5) was added to 15 ml semi-soft MHB agar (0.75% w/v agar), which was overlaid on 45 ml MHB agar (1.5% w/v agar). After the agar had solidified, antibiotic disks from OXOID, United Kingdom were placed on top of the lawn of cells. Ciprofloxacin (CIP 5 μg), norfloxacin (NOR 10 μg), moxifloxacin (MXF 5 μg), neomycin (N 30 μg), netilmicin (NET 30 μg), amikacin (AK 30 μg), cefepime (FEP 30 μg), ceftriaxone (CRO 30 μg), cefoperazone (CFP 75 μg), cefoperazone/sulbactam 2:1 (SCF 105 μg) fosfomycin (FOS 30 μg), and tetracycline (TE 30 μg) were tested. The plates were incubated overnight at 37°C before the inhibition zones were measured (Schwalbe et al., 2007).

### Determination of Antibiotic Minimal Inhibition Concentrations (MICs)

Minimal inhibition concentrations of antibiotics, paraquat, and ethidium bromide against bacterial strains were measured using a broth microdilution method. A 2-fold dilution series of each test chemical was made in MHB as 100 μl aliquots in the wells of a 96-well plate and 10 μl of bacterial culture was added to each. The cultures used were diluted from overnight cultures in MHB adjusted to OD_600__*nm*_ of 0.01. The plates were incubated overnight at 37°C when OD_600__*nm*_ was measured using a microplate reader (EPOCH 2). The MIC was defined as the lowest concentration where bacterial growth was inhibited (Coyle, 2005; Vattanaviboon et al., 2018).

### Gene Expression Analysis

Expression of the genes of interest was measured using semi-quantitative RT-PCR. Overnight cultures of strains in MHB with selection as appropriate were sub-cultured in MHB with an initial OD_600__*nm*_ of 0.1. Log phase (OD_600__*nm*_ of 0.4–0.6) cells were used in all experiments. For stress induction studies, 0.02% of NaOCl; 100 μM of FeCl_3_; 100 μM 2,2′-Bipyridine (Dipy); 500 μM of diamide (DM); 100 μM of NEM; 250 μM of plumbagin (PB), 1 mM of menadione (MD), and 1 mM of paraquat (PQ); 1 mM of hydrogen peroxide (H_2_O_2_); 500 μM of cumene hydroperoxide (CHP) and 500 μM of t-butyl hydroperoxide (tBH) were included in the log phase cells in LB medium for 15 min before pelleting the cells, as previously (Romsang et al., 2018). RNA was prepared using a hot acid phenol method. The contaminated DNA in RNA samples was removed by DNase I treatment, following the manufacturer’s instructions (Thermo Scientific). 1 μg treated RNA was used as the template for a reverse transcription reaction to synthesize cDNA using random hexamer primers (Thermo Scientific). The cDNA was used as a template for gene expression analysis using SYBR^®^ FAST qPCR kit (KAPA Biosystems) running in Applied Biosystems StepOnePlus thermal cycler with the gene specific primers for *mfs1* (BT5958 and BT5960), *mfs2* (BT5961 and BT5963), *mexX* (BT6223 and BT6224), *mexZ* (BT6417 and BT6418), *armZ* (BT7158 and BT7159), *oprM* (BT6304 and BT6305), *htpX* (BT7164 and BT71655), *PA5528* (BT7162 and BT7163), *amgR* (BT6423 and BT6424), *amgS* (BT6526 and BT6527), and *16S rRNA* (BT2781 and BT2782) (Supplementary Table 1). Relative expression (2^–ΔΔCt^) was calculated using *16S rRNA* as an internal control from StepOne software and was expressed as fold-change relative to control. Statistical analysis was preformed from three biologically independent experiments.

### Proteomics Analysis of Envelope Proteins

Overnight cultures in MHB with antibiotic selection as required were sub-cultured into fresh MHB broth with selection and incubated until bacterial growth reached late log phase (OD_600__*nm*_ of 0.6–0.8). The cells were pelleted by centrifugation at 4,000 rpm at 4°C for 15 min. The pellets were resuspended in 30 mM Tris–HCl, pH 8.0 then sonicated 1 s on, 1 s off for 3 min with 63% amplitude using a Sonics Vibracell VC-505TM (Sonics and Materials Inc., Newton, CT, United States). Cell lysates were centrifuged at 10,000 rpm at 4°C for 30 min to remove cell debris. The supernatant was recentrifuged at 20,000 rpm at 4°C for 60 min to pellet envelope proteins. The pellets were air-dried and resuspended in 30 mM Tris–HCl, pH 8.0 containing 0.5% w/v SDS. 5 μg of proteins was loaded onto 11% w/v SDS-PAGE and run until the dye-front reach 1 cm into the separating gel. Proteins within the gels were stained with Instant Blue Protein Stain (Expedeon), cut and subjected to in-gel tryptic digestion (ProGest automated digestion unit, Digilab, United Kingdom). Solubilized peptides in 1% v/v formic acid were fractionated using an Ultimate 3000 nano HPLC system connected with an LTQ-Orbitrap Velos mass spectrometer (Thermo Scientific). Peptides were separated by Acclaim PepMap C18 nano-trap column (Thermo Scientific) with gradient mobile phase of 0.1% v/v formic acid in water and 80% v/v acetonitrile with 0.1% v/v formic acid. The data were processed and quantified using Proteome Discoverer software v1.2 (Thermo Scientific) and searched against *P. aeruginosa* strain PAO1 (ATCC15692) database, which contains 5,563 proteins using the SEQUEST (Ver. 28 Rev. 13) algorithm. Data from three biological replicates were calculated for relative quantity compared to the control strain as previously described (Jiménez-Castellanos et al., 2018).

### Statistical Analysis

Paired *t***-**tests were used for general statistical analysis. *p-*value < 0.05 was considered significant and indicated with an asterisk (^∗^). For disk diffusion assay, a Mann–Whitney *U* test was applied.

## Results and Discussion

### Gene Expression Analysis of *mfs1* and *mfs2* Under Stress Conditions

**FIGURE 1:**
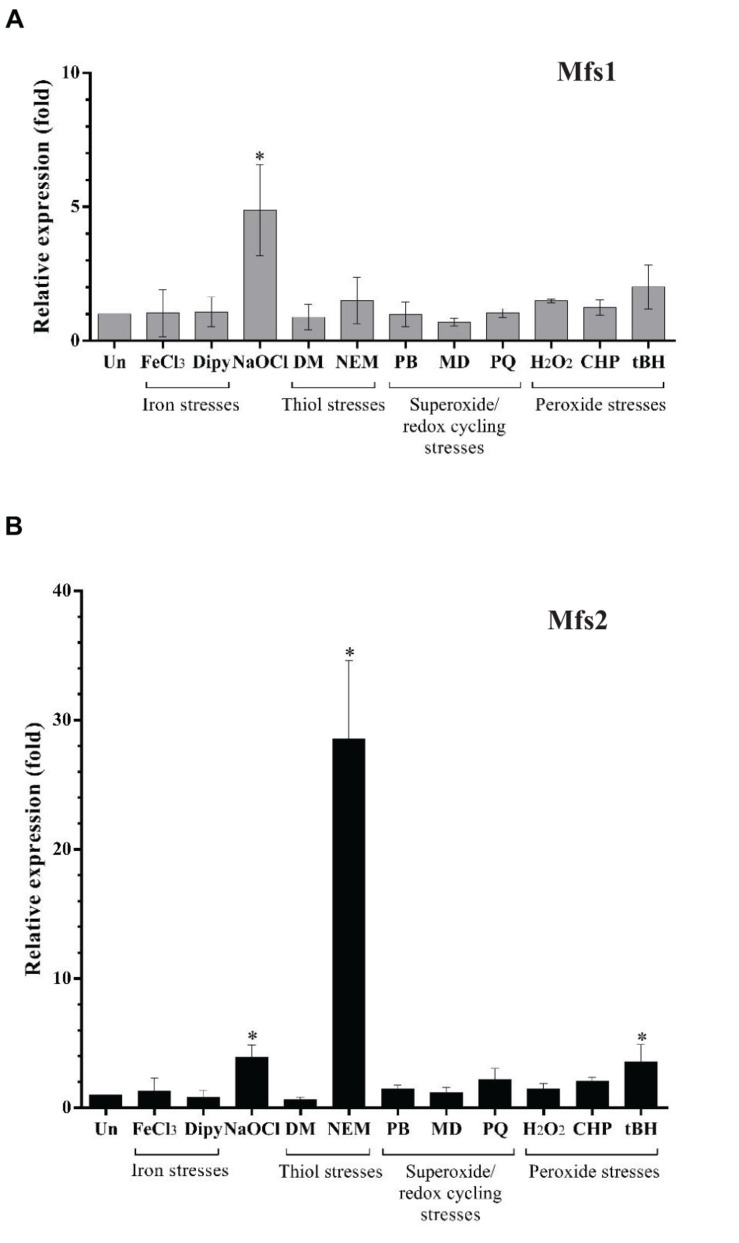
Relative expression of **(A)** *mfs1* and **(B)** *mfs2* in response to stresses in PAO1 wild-type, determined by semi-quantitative real-time RT-PCR. Mid-log phase PAO1 wild-type cells were treated with 100 μM of iron (FeCl_3_), 100 μM of iron depletion agent, 2,2′-Bipyridine (Dipy), 0.02% of NaOCl, 500 μM of diamide (DM), 100 μM of N-Ethylmaleimide (NEM), 250 μM of plumbagin (PB), 1 mM of menadione (MD) 1 mM of paraquat (PQ), 1 mM of hydrogen peroxide (H_2_O_2_), 500 μM of cumene peroxide (CHP), and 500 μM of t-butyl hydroperoxide (tBH) for 15 min. After normalizing data with internal control (16S rRNA), gene expression in each condition was compared to the untreated control (Un) and fold changes were calculated. Data presented are means ± SD of three biological replicates. Asterisk (^∗^) indicates significant difference relative to untreated (Un) (*p-*value < 0.05).

Our secondary analysis of microarray data (Small et al., 2007) for *P*. *aeruginosa* undergoing hypochlorite stress indicated that *mfs1* and *mfs2* expression was increased by 10**-**fold and 5**-**fold, respectively. To confirm and extend this finding, *mfs1* and *mfs2* expression in response to NaOCl and other stresses such as oxidative stresses, iron stresses and thiol stresses were measured using semi-quantitative real-time RT-PCR. Expression of both *mfs1* and *mfs2* was increased upon NaOCl stress by 5-fold. In addition, *mfs2* was upregulated following treatment with NEM, an electrophile and thiol depleting agent (Vattanaviboon et al., 2001; Reisz et al., 2013) by 30-fold and by 5-fold following treatment with tBH (Figure 1). Apart from the increased expression of *mfs1* and *mfs2* in response to hypochlorite stress, secondary analysis of the microarray data also showed that the expressions of other known antibiotic resistance-associated genes *mexAB-oprM, mexXY*, *mexZ, armZ*, and *amgRS* were 4, 3, 2, 10, and 4-fold higher than in untreated cells (Small et al., 2007).

### The Importance of Mfs1 and Mfs2 in Antibiotic Susceptibility

#### The Over-Expression of *mfs1* and *mfs2* Reduces Antibiotic Susceptibility

**FIGURE 2:**
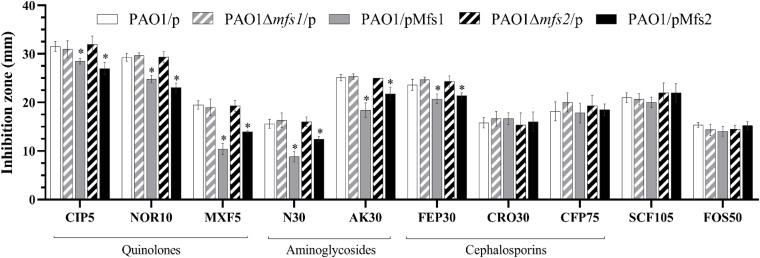
Antibiotic susceptibility against the fluoroquinolones, ciprofloxacin (CIP, 5 μg in disk), norfloxacin (NOR 10 μg), moxifloxacin (MXF 5 μg); aminoglycosides, neomycin (N 30 μg), amikacin (AK 30 μg); cephalosporins, cefepime (FEP 30 μg), ceftriaxone (CRO 30 μg), cefoperazone (CFP 75 μg); and cefoperazone/sulbactam 2:1 (SCF 105 μg), and fosfomycin (FOS 30 μg) for PAO1/p, PAO1Δ*mfs1*/p, PAO1/pMfs1, PAO1Δ*mfs2*/p, and PAO1/pMfs2. Data presented are means ± standard deviations of three biological replicates. Asterisk (^∗^) indicates significant difference relative to PAO1/p (*p-*value < 0.05).

To investigate whether increased expression of *mfs1* and/or *mfs2* affected antibiotic susceptibility, each gene was over-expressed by introducing recombinant plasmids pMfs1 or pMfs2, into PAO1. Assays of susceptibility, quantified by measuring growth inhibition zone diameters around antibiotic disks, revealed that both PAO1/pMfs1 and PAO1/pMfs2 were less susceptible to fluoroquinolones; ciprofloxacin, norfloxacin, and moxifloxacin and aminoglycosides; neomycin and amikacin; and cefepime relative to plasmid only control (PAO1/p) (Figure 2). Changes in susceptibility were confirmed by measuring MICs of antibiotics (Table 1). The MIC of neomycin against PAO1/pMfs1 or PAO1/pMfs2 increased 16-fold or 2-fold relative to PAO1/p. MICs of gentamicin and moxifloxacin increased 4-fold against PAO1/pMfs1 and 2-fold against PAO1/pMfs2 relative to PAO1/p. Other 2-fold increases in MIC were seen for other agents against one or both recombinants (Table 1), showing consistency with the disk susceptibility test data.

**TABLE 1:** MIC values against PAO1/pMfs1 and PAO1/pMfs2 of aminoglycosides, quinolones, cephalosporins, paraquat, and ethidium bromide.

**MIC (μg/ml)**			
**Antibiotics**	**PAO1/p**	**PAO1/pMfs1**	**PAO1/pMfs2**
Neomycin	4	64	8
Gentamicin	1	4	2
Tobramycin	0.5	1	0.5
Ciprofloxacin	0.125	0.25	0.25
Norfloxacin	0.25	0.5	0.5
Levofloxacin	0.5	1	1
Moxifloxacin	0.5	2	1
Cefepime	2	4	4
Ceftazidime	2	4	2
Paraquat	300	450	500
Ethidium Bromide	1,024	1,024	2,048

*The value reported is the mode of seven independent experiments.*

The MFS transporter family is large and members are involved in the extrusion of various types of substrates (Srijaruskul et al., 2015). BLASTP (Altschul et al., 1997) analysis (Supplementary Table 2) revealed that Mfs1 and Mfs2 share 21.4% and 37.6% identity with SmvA, respectively, which is known to extrude paraquat or methyl viologen (Santiviago et al., 2002). Accordingly, to investigate the possibility that Mfs1 and/or Mfs2 can provide protection against paraquat, MICs of paraquat were determined. This showed that PAO1/pMfs1 and PAO1/pMfs2 strains were 1.5-fold (450 μg/mL) and 1.7-fold (500 μg/mL) less susceptible to paraquat than PAO1/p (300 μg/mL). This suggests that Mfs1 and Mfs2 can extrude paraquat. Moreover, the MIC of ethidium bromide against PAO1/pMfs2 (only) was 2-fold greater than against PAO1/p (Table 1). Interestingly, Mfs2 is 19.8% identical to QacA (Supplementary Table 2), which is a quaternary ammonium compounds and ethidium bromide pump.

Accordingly, we conclude that over-production of Mfs1 and Mfs2 is associated with reduced susceptibility to multiple antibiotics, paraquat and ethidium bromide in *P. aeruginosa* PAO1. It may be that Mfs1 and Mfs2 over-production have other phenotypic effects in PAO1, but that remains to be seen.

#### The Disruption of *mfs1* or *mfs2* Does Not Affect Antibiotic or Paraquat Susceptibility

Given the possible roles of Mfs1 and Mfs2 as multi-drug transporters, the genes were disrupted in PAO1. However, there was no significant difference in antibiotic susceptibility in the mutants (PAO1Δ*mfs1*/p and PAO1Δ*mfs2*/p) relative to the PAO1/p (Figure 2). Importantly, in deletion mutants complemented with the same *mfs1* and *mfs2* expression plasmid used previously (PAO1Δ*mfs1*/pMfs1 and PAO1Δ*mfs2*/pMfs2), very similar reductions in antibiotic susceptibility to those seen in wild-type PAO1 carrying the plasmids were observed (data not shown).

It is possible that the disruption of *mfs1* or *mfs2* does not affect antibiotic susceptibility is that the pumps are not produced at significant enough levels in wild-type cells for disruption to have a phenotypic effect. Ideally, to prove whether Mfs1 and Mfs2 have the capacity to pump antibiotics, they should be over-produced in a *P. aeruginosa* background lacking all major antibiotic efflux pumps (Morita et al., 2001). However, such a strain was not available to us, so we chose to over-produce the pumps in *E. coli* DH5α, an antibiotic susceptible laboratory strain. This strain does produce the AcrAB-TolC antibiotic efflux pump, and it would be better to have used a derivative lacking major efflux pumps such as Kam3 (Morita et al., 1998) and/or Kam32 (Chen et al., 2002) but this background is less likely to mask the effect of Mfs1 and Mfs2 on antibiotic susceptibility than using *P. aeruginosa* PAO1. There were no significant changes in antibiotic susceptibilities following over-production of Mfs1 and Mfs2 in *E. coli* DH5α (Supplementary Figure 1). So whilst we cannot entirely exclude the possibility that Mfs1 and/or Mfs2 efflux antibiotics to some degree, clearly this activity is weak at best.

Susceptibility of PAO1Δ*mfs1* and PAO1Δ*mfs2* to paraquat was also not significantly different from wild-type (Supplementary Figure 2). This may also be due to low wild-type expression levels, but it remains possible that Mfs1 and Mfs2 are paraquat pumps, and that the result we observed is due to redundancy among paraquat protection mechanisms in *P. aeruginosa*. To date, however, no paraquat efflux pumps have been characterized in *P. aeruginosa*. A pump encoded by a chromosomal gene homologous to *emrE* does pump out paraquat when reconstituted in lipid vesicles (Ninio et al., 2001), but disruption of this gene, like we found with *mfs1* and *mfs2*, did not increase susceptibilioty to paraquat in *P. aeruginosa* (Li et al., 2003). We tried to identify paraquat transporters by searching the PAO1 genome for homologs of known paraquat pumps including *Salmonella enterica* subsp. enterica serovar Typhimurium SmvA (Santiviago et al., 2002), *Stenotrophomonas maltophilia* MfsA (Srijaruskul et al., 2015) and *Staphylococcus aureus* QacA (Tennent et al., 1989) using BLASTP (Altschul et al., 1997), but only Mfs2 shares significant identity (37.6%) with SmvA (Supplementary Table 2). However, PAO1 also has several antioxidant enzymes that play important roles in paraquat detoxification such as glucose-6-phosphate dehydrogenase (Ma et al., 1998) and superoxide dismutase (Hassett et al., 1995). Therefore, the presence of these systems may explain why paraquat susceptibility was not increased in PAO1Δ*mfs1* and PAO1Δ*mfs2* relative to wild-type.

### Envelope Proteomics Analysis of in PAO1/pMfs1 and PAO1/pMfs2

Changes in antibiotic susceptibility of PAO1/pMfs1 and PAO1/pMfs2 were similar yet these over-produced proteins share only 22.5% identity. We have demonstrated that antibiotics are not significant Mfs1 and Mfs2 substrates when produced in *E. coli*, so we hypothesized that over-production of the either pump leads to reduced antibiotic susceptibility through activation of a common or indirect mechanism. To characterize this mechanism, proteomics analysis of envelope proteins was undertaken. The mass spectrometry proteomics data have been deposited to the ProteomeXchange Consortium via the PRIDE (Perez-Riverol et al., 2019) partner repository with the dataset identifier PXD021066. Using at least a 4-fold statistically significant change in protein abundance relative to PAO1/p as a cut-off, PAO1/pMfs1 and PAO1/pMfs2 shared 30 differentially regulated proteins in common (11 upregulated and 19 downregulated) (Table 2). Among these 11 upregulated proteins were subunits of the known RND-type antibiotic efflux pump, MexXY. MexX and MexY production was increased by 71-fold and 190-fold in PAO1/pMfs1 and by 29-fold and 102-fold in PAO1/pMfs2, respectively. It is known that MexXY over-production reduces susceptibility to quinolones, macrolides, tetracyclines, lincomycin, chloramphenicol, aminoglycosides, and some β-lactams (Masuda et al., 2000; Poole, 2012). This known substrate profile matches well with the susceptibility changes seen in PAO1/pMfs1 and PAO1/pMfs2 relative to PAO1/p (Figure 2).

**TABLE 2:** List of significant protein abundance changes observed in PAO1**/**pMfs1 and PAO1**/**pMfs2 relative to PAO1**/**p.

**Accession**	**Description**		**Fold-change in PAO1/pMfs1 vs. PAO1/p**	***p-*value of PAO1/pMfs1 vs. PAO1/p**	**Fold-change in PAO1/pMfs2 vs. PAO1/p**	***p-*value of PAO1/pMfs2 vs. PAO1/p**
G3XCW2	Resistance-Nodulation-Cell Division (RND) multidrug efflux transporter	MexY	190.16	0.001	102.11	0.001
G3XD21	Resistance-Nodulation-Cell Division (RND) multidrug efflux membrane fusion protein	MexX	70.58	0.000	28.76	0.006
G3XDA8	Phosphate-binding protein PstS	PstS	<0.05	0.000	<0.05	0.000
P24474	Nitrite reductase	NirS	<0.05	0.001	<0.05	0.001
P38100	Carbamoyl-phosphate synthase large chain	CarB	<0.05	0.009	<0.05	0.009
P53593	Succinate–CoA ligase [ADP-forming] subunit beta	SucC	<0.05	0.003	0.10	0.005
Q51422	Aspartate–tRNA(Asp/Asn) ligase	AspS	<0.05	0.019	0.19	0.049
Q51567	Succinate–CoA ligase [ADP-forming] subunit alpha	SucD	<0.05	0.002	<0.05	0.002
Q9HTD0	Probable biotin carboxylase subunit of a transcarboxylase	PA5436	<0.05	0.027	<0.05	0.027
Q9HVI7	Serine hydroxymethyltransferase 3	GlyA2	<0.05	0.000	0.15	0.002
Q9HVK2	Uncharacterized protein	PA4582	>20	0.002	>20	0.004
Q9HX11	Uncharacterized protein	PA4016	0.19	0.002	<0.05	0.000
Q9HXR2	Uncharacterized protein	PA3730	>20	0.001	>20	0.001
Q9HZ76	UDP-2-acetamido-2-deoxy-3-oxo-D-glucuronate aminotransferase	WbpE	<0.05	0.010	0.16	0.019
Q9HZG8	Uncharacterized protein	PA3040	<0.05	0.006	0.12	0.014
Q9I0L1	High frequency lysogenization protein HflD homolog	HflD	10.30	0.001	5.39	0.008
Q9I0L5	Isocitrate dehydrogenase [NADP]	Icd	<0.05	0.000	0.16	0.007
Q9I2W9	Phosphoenolpyruvate synthase	PpsA	<0.05	0.002	<0.05	0.002
Q9I314	Type III export protein PscJ	PscJ	11.57	0.002	6.07	0.010
Q9I3F5	Aconitate hydratase A	AcnA	<0.05	0.000	<0.05	0.000
Q9I3L9	Sulfate-binding protein of ABC transporter	CysP	<0.05	0.002	<0.05	0.002
Q9I3Q1	Uncharacterized protein	PA1450	>20	0.001	>20	0.001
Q9I427	Cytochrome bo(3) ubiquinol oxidase subunit 2	CyoA	<0.05	0.003	<0.05	0.003
Q9I513	Phosphoribosylformylglycinamidine cyclo-ligase	PurM	<0.05	0.004	0.24	0.035
Q9I528	Two-component sensor	PA0930	>20	0.031	>20	0.001
Q9I5R3	Probable short-chain dehydrogenase	PA0658	>20	0.001	>20	0.000
Q9I5Y1	Fructose-bisphosphate aldolase	Fba	<0.05	0.004	<0.05	0.004
Q9I631	Probable molybdenum transport regulator	PA0487	<0.05	0.007	<0.05	0.007
Q9I748	Uncharacterized protein	PA0084	4.66	0.011	16.95	0.018
Q9I759	Probable ATP-binding component of ABC transporter	PA0073	7.32	0.011	5.22	0.018

*Significant changes are defined as *p****-***value < 0.05 and fold**-**change > 4 or < 0.25. All proteomic data are available in Supplementary Material.*

To determine whether over-production of another unrelated MFS transporter increases *mexX* expression and reduces antibiotic susceptibility, PAO1 harboring pTetA(C) for over-expression of *tetA*(C) (Leesukon et al., 2013) was used in the experiments. The tetracycline resistance gene, *tetA*(C), encodes an MFS efflux pump containing 12 transmembrane domains that transport tetracycline across the inner membrane (Schnappinger and Hillen, 1996). As shown in Supplementary Figure 3A, the expression of *tetA*(C) rendered PAO1 resistant to tetracycline judging from disk diffusion confirming that *tetA*(C) was expressed. We found that over-expression of *tetA*(C) did neither significantly induce *mexX* expression (Supplementary Figure 3B) nor alter the susceptibility of PAO1 to ciprofloxacin, a MexXY substrate (Supplementary Figure 3A). Thus, the enhanced expression of *mexX* following Mfs1 and Mfs2 over-production is not universal effect of MFS protein over-production.

**FIGURE 3:**
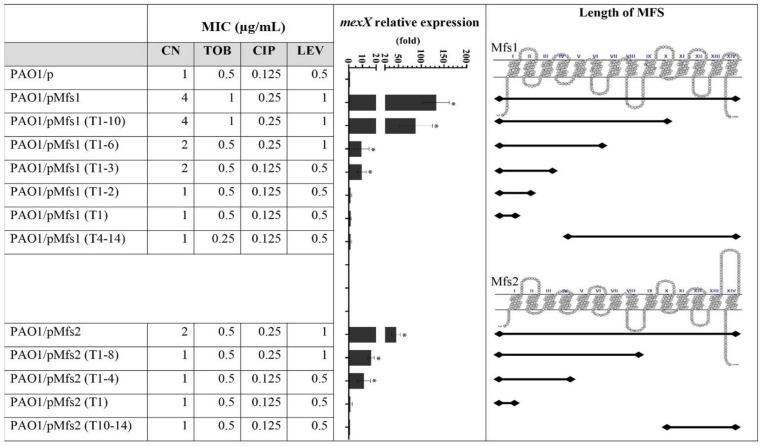
Expression of *mexX* and aminoglycoside [gentamicin (CN) and tobramycin (TOB)] or quinolone [ciprofloxacin (CIP) and levofloxacin (LEV)] MICs against PAO1**/**pMfs1 and PAO1**/**pMfs2 and various truncated derivatives. Mfs1 and Mfs2 membrane topology were generated from SOSUI (Hirokawa et al., 1998; Mitaku and Hirokawa, 1999; Mitaku et al., 2002). MICs data shown are the modes of seven independent experiments. Gene expression analysis data are present in means ± SD of three biological replicates. Asterisk (^∗^) indicates significant difference relative to PAO1**/**p (*p*-value < 0.05).

We next tested whether over-producing functionally inactive, truncated Mfs1 and Mfs2 proteins increased MexXY production. To do this, we sub-cloned *mfs1* which encodes a 14-transmembrane spanning MFS (wild-type) in as a truncated form that encoded a 10-transmembrane spanning MFS (T1–10), Both PAO1/pMfs1 and PAO1/pMfs1 (T1–10) had similar antibiotic susceptibility (Figure 3). Proteomics analysis of PAO1/pMfs1 (T1–10) showed that MexX and MexY abundances were 7-fold and 6-fold higher, respectively, relative to PAO1/p. These data support the hypothesis that Mfs1 is not an antibiotic transporter, but that the effect of its over-production on antibiotic susceptibility is indirect. Furthermore, that Mfs1 does not need to be functional as a transporter to have this effect.

To characterize the transmembrane domains of Mfs1 and Mfs2 that are important for this effect, a subcloning approach, where parts of full-length of MFS proteins were deleted, was performed. Subcloning of *mfs1* generated six different Mfs1 derivatives consisting of Mfs1 (T1–10), Mfs1 (T1–6), Mfs1 (T1–3), Mfs1 (T1–2), Mfs1 (T1), and Mfs1 (T4–11). The subclones are named according to number of transmembrane domains remaining, for example, Mfs1 (T4–11) is consisted of the 4th to the 11th transmembrane domains. For *mfs2*, four subclones were constructed, i.e., Mfs2 (T1–8), Mfs2 (T1–4), Mfs2 (T1), and Mfs2 (T10–14). The membrane topologies of the truncated Mfs1 and Mfs2 proteins are shown in Figure 3. Once these various recombinant plasmids were introduced into PAO1, data revealed that Mfs1 required a minimum of three transmembrane segments to increase *mexX* expression and reduce antibiotic susceptibility (i.e., truncations leaving either the first four or the last three segments intact gave this effect). For Mfs2, the minimal effect was with the first four transmembrane segments to increase *mexX* expression, but the first eight transmembrane segments were required to increase it sufficiently to reduce antibiotic susceptibility (Figure 3).

### Mfs1/Mfs2 Over-Production Activates *mexXY* Expression in a Mechanism Dependent on *armZ*

Regulation of *mexXY* expression is complex. MexZ is a TetR-type local repressor, but other regulators are involved (Matsuo et al., 2004; Yamamoto et al., 2009). Associations have been made between *mexXY* expression and oxidative stress, ribosomal stress from ribosome targeted antibiotics such as tetracycline, chloramphenicol, erythromycin, streptomycin, and aminoglycosides (Jeannot et al., 2005; Morita et al., 2006; Poole, 2012). However, *in vitro* data have shown that MexZ does not bind these agents (Matsuo et al., 2004). In fact, expression of *armZ* is increased in response to oxidative stress, ribosome targeted drugs, and ribosomal induced stresses (Morita et al., 2006; Yamamoto et al., 2009; Fraud and Poole, 2011; Hay et al., 2013; Kawalek et al., 2019) and ArmZ binds MexZ reducing its ability to repress *mexXY* expression (Morita et al., 2006; Hay et al., 2013; Li et al., 2015). Additionally, AmgRS is a two-component system, which responds to aberrant polypeptides that disrupt the inner membrane generating envelope stress (Lau et al., 2015). Activating of AmgRS system drives expression of the *htpX* and *PA5528* genes and this promotes expression of *mexXY* via increasing the ArmZ-MexZ interaction (Lau et al., 2015).

**FIGURE 4:**
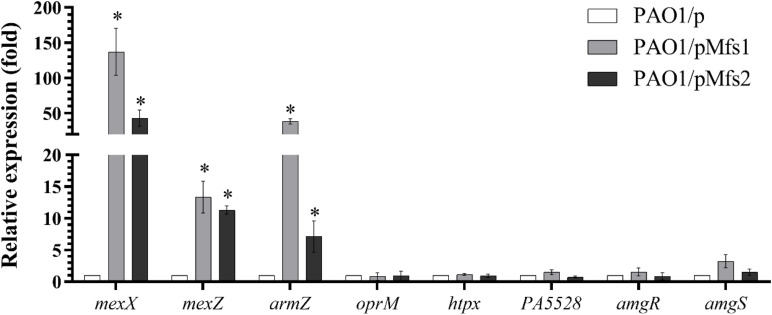
Expression analysis of *mexX* and genes involved in *mexXY* regulation in PAO1/pMfs1 and PAO1/pMfs2 using semi-quantitative RT-PCR compared to PAO1/p. Data shown are mean ± SD and asterisk (^∗^) indicates significant difference relative to PAO1/p (*p-*value < 0.05).

To test which, if either, of these regulatory systems are involved in Mfs1 and Mfs2 mediated changes in MexXY production, additional gene expression analysis was performed. These data confirmed that *mexX* is over-expressed in PAO1/pMfs1 (150-fold), PAO1/pMfs1 (T1–10) (90-fold) and PAO1/pMfs2 (50-fold), relative to PAO1/p (Figures 3, 4). Moreover, expression of *mexZ* and *armZ* were both also increased in PAO1/pMfs1 (13-fold and 40-fold, respectively) and PAO1/pMfs2 (11-fold and 7-fold, respectively) (Figure 4). No significant changes were observed in expression of *oprM* (an unlinked gene encoding the outer membrane protein that partners with MexXY), *htpX, PA5528, amgR* or *amgS* in PAO1/pMfs1 or PAO1/pMfs2 relative to PAO1/p (Figure 4).

**FIGURE 5:**
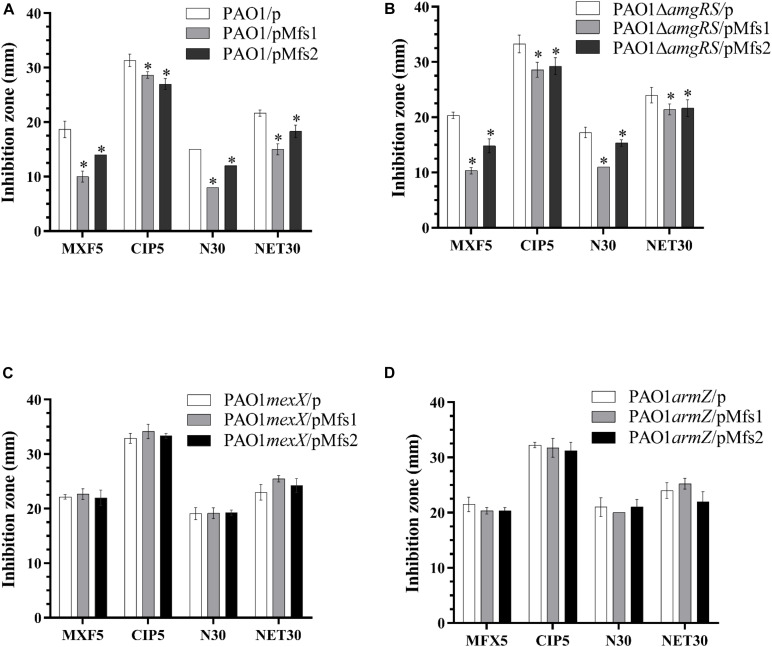
Antibiotic susceptibility profiles of in PAO1, PAO1Δ*amgRS*, PAO1*mexX* and PAO1*armZ* carrying pMfs1 and pMfs2. **(A)** PAO1/p, PAO1/pMfs1. PAO1/pMfs2, **(B)** PAO1Δ*amgRS*/p, PAO1Δ*amgRS*/pMfs1, PAO1Δ*amgRS*/pMfs2, **(C)** PAO1*mexX*/p, PAO1*mexX*/pMfs1, PAO1*mexX*/pMfs2, **(D)** PAO1*armZ*/p, PAO1*armZ*/pMfs1, and PAO1*armZ*/pMfs2 against moxifloxacin (MFX, 5 μg in disk), ciprofloxacin (CIP 5 μg), neomycin (N 30 μg), and netilmicin (NET 30 μg) by using the Kirby-Bauer method. Data presented are means ± SD of three biological replicates. Asterisk (^∗^) indicates significant difference relative to PAO1/p, PAO1Δ*amgRS*/p, PAO1*mexX*/p, and PAO1*armZ*/p, respectively (*p-*value < 0.05).

To further study the regulatory processes involved, *mexX, armZ*, and *amgRS* mutants were constructed in PAO1. The result showed that over-production of Mfs1 and Mfs2 in PAO1*mexX* and PAO1*armZ* mutants has no significant effect on antibiotic susceptibility, relative to the control, PAO1*mexX/*p and PAO1*armZ/*p, respectively, but that reduced susceptibility was seen in the PAO1Δ*amgRS* mutant upon over-production of Mfs1 or Mfs2 (Figure 5). We therefore conclude that over-production of Mfs1 and Mfs2 triggers transcription of *armZ* and that ArmZ modulates MexZ, de-repressing *mexXY* expression, which is the ultimate cause of reduced antibiotic susceptibility. We found no evidence for the AmgRS regulatory element being involved in this process.

There was no difference between the MICs of ciprofloxacin, levofloxacin, gentamicin, and amikacin against PAO1 in the presence and absence of NaOCl, an inducer of *mfs1* and *mfs2* expression (Supplementary Table 3). We conclude, therefore, that to see a reduction in antibiotic susceptibility via this *armZ*-*mexZ*-*mexXY* cascade, *mfs1* and *mfs2* expression must be high, as seen upon carriage in the multicopy plasmid used for this work.

### Reduced Paraquat Susceptibility Following Mfs1 and Mfs2 Over-Production Is *mexX* and *armZ* Independent but Dependent on Mfs1/Mfs2 Functionality

Over-production of Mfs1 and Mfs2 reduces paraquat susceptibility (Table 1), so we investigated whether *mexX* and *armZ* are necessary for this phenotype using a disk diffusion method. This revealed that paraquat susceptibility reduced even in PAO1*mexX/*pMfs1 and PAO1*mexX/*pMfs2 compared to PAO1*mexX/*p and in PAO1*armZ/*pMfs2 compared to PAO1/p and PAO1*mexX/*p (Supplementary Figure 2). Finally, we found that reduced paraquat susceptibility did not occur on over-production of any truncated version of Mfs1 and Mfs2 (Supplementary Figure 4). Hence reduced paraquat susceptibility is directly associated with Mfs1 and Mfs2 over-production, and we therefore propose that they function as paraquat transporters in *P. aeruginosa*.

## Conclusion

Hypochlorite stress causes over-production of Mfs1 and Mfs2. We conclude that this reduces paraquat susceptibility because this is a direct substrate for these novel MFS transporters. The reason for this conclusion is that over-production of truncated version of these proteins does not reduce paraquat susceptibility. Final proof must await *in vitro* efflux assays, as performed previously for a *P. aeruginosa* protein related to EmrE (Ninio et al., 2001). In addition, over-production of Mfs1 and Mfs2 decreased antibiotic susceptibility, particularly for aminoglycosides, quinolones and some cephalosporins. This is not directly caused by the MFS transporters because truncation of the proteins did not stop reduced antibiotic susceptibility, so long as just a small number of transmembrane segments were left intact. In fact, the mechanism by which Mfs1 and Mfs2 over-production leads to antibiotic susceptibility is by increasing ArmZ production, leading to de-repression of *mexXY* transcription, which is known to be an efflux pump able to transport quinolones, aminoglycosides and cephalosporins. This mechanism is independent from *amgRS* since reduced antibiotic susceptibility is observed upon over-production of Mfs1 and Mfs2 even if *amgRS* has been disrupted. We do not know the mechanism by which Mfs1 and Mfs2 over-production activates MexXY production, but it is possible that enhanced translation of the hydrophobic nascent polypeptides of Mfs1 of Mfs2, or truncated fragments thereof, causes ribosome stalling due to adhesion to the ribosomal exit tunnel (Woolstenhulme et al., 2013), a ribosomal stress known to activate *armZ* expression (Lau et al., 2015), thereby derepressing the expression of *mexXY.* We could find no reports in the literature showing MFS pump over-production activating antibiotic resistance via a similar mechanism, but it is possible that this is a more general phenomenon. It is not universal, however, since over-production of TetA(C) did not increase *mexXY* expression. One important implication of this work is that if paraquat use were to select mutants that over-produce Mfs1 or Mfs2, due to their reduced susceptibility to this chemical, the collateral damage of this would reduce antibiotic susceptibility in what is already a difficult to treat human pathogen.

## Data Availability Statement

The original contributions presented in the study are publicly available. This data can be found here: https://www.ebi.ac.uk/pride/archive/projects/PXD021066.

## Author Contributions

SM conceived and designed the experiments. PD and NS performed the experiments. PD, NC, PV, and SM analyzed and interpreted the data. PD wrote the first draft of the manuscript. SM, MA, PV, and NC edited the manuscript. All authors contributed to manuscript revision, read, and approved the submitted version.

## Conflict of Interest

The authors declare that the research was conducted in the absence of any commercial or financial relationships that could be construed as a potential conflict of interest.
