# Supplementary material for: Over-Expression of Hypochlorite Inducible Major Facilitator Superfamily (MFS) Pumps Reduces Antimicrobial Drug Susceptibility by Increasing the Production of MexXY Mediated by ArmZ in Pseudomonas aeruginosa
Source: Front Microbiol. 2021 Jan 12;11:592153. doi: 10.3389/fmicb.2020.592153 (PMC7835679; doi:10.3389/fmicb.2020.592153)
Supplement: Supplementary file 1 [file Data_Sheet_1.docx]

| **Table S1.** List of bacterial strains, plasmids and primers used in the studies. | | |
| --- | --- | --- |
| **Strains/plasmids/ primers** | **Description** | **Reference** |
| **Strains**  ***E. coli*** | | |
| DH5α | F^–^ φ80*lac*ZΔM15 Δ(*lac*ZYA-*arg*F)U169 *rec*A1 *end*A1 *hsd*R17 (r_K_^–^, m_K_^+^) *pho*A *sup*E44 λ^–^ *thi*-1 *gyr*A96 *rel*A1/F′ [*pro*AB^+^ *lac*I^q^ZΔM15 *zzf*::*Tn*5(Km^r^)] | Stratagene Inc. |
| BW20767 | RP4-Tc-::Mu-1, kan::Tn7 integrant, *leu*63::IS10, *recA*1, *zbf*-5, *creB*510, *hsd*R17 *endA*1, *thi*, *uidA* (ΔMluI)::*pir^+^* | (Marx and Lidstrom, 2002) |
| ***P. aeruginosa*** | | |
| PAO1 | ATCC15692 |  |
| PAO1/p | PAO1 harbouring pBBR1MCS-4, Ap^r^ | This study |
| PAO1/pMfs1 (T1-14) | PAO1 harbouring pMfs1, Ap^r^ | This study |
| PAO1/pMfs2 (T1-14) | PAO1 harbouring pMfs2, Ap^r^ | This study |
| PAO1∆*mfs1* | PAO1 with *PA1262* deletion | This study |
| PAO1∆*mfs1*/p | PAO1∆*mfs1* harbouring pBBR1MCS-4, Ap^r^ | This study |
| PAO1∆*mfs1*/pMfs1 | PAO1∆*mfs1* harbouring pMfs1, Ap^r^ | This study |
| PAO1∆*mfs2* | PAO1 *PA1282* deletion | This study |
| PAO1∆*mfs2*/p | PAO1∆*mfs2* harbouring pBBR1MCS-4, Ap^r^ | This study |
| PAO1∆*mfs2*/pMfs2 | PAO1∆*mfs2* harbouring pMfs2, Ap^r^ | This study |
| PAO1/pMfs1 (T1-10) | PAO1 harbouring pMfs1 containing 10-transmembrane, Ap^r^ | This study |
| PAO1/pMfs1 (T1-6) | PAO1 harbouring pMfs1 containing 6-transmembrane, Ap^r^ | This study |
| PAO1/pMfs1 (T1-3) | PAO1 harbouring pMfs1 containing 3-transmembrane, Ap^r^ | This study |
| PAO1/pMfs1 (T1-2) | PAO1 harbouring pMfs1 containing 2-transmembrane, Ap^r^ | This study |
| PAO1/pMfs1 (T1) | PAO1 harbouring pMfs1 containing 1-transmembrane, Ap^r^ | This study |
| PAO1/pMfs1 (T4-14) | PAO1 harbouring pMfs1 containing 11-transmembrane, Ap^r^ | This study |
| PAO1/pMfs2 (T1-8) | PAO1 harbouring pMfs2 containing 8-transmembrane, Ap^r^ | This study |
| PAO1/pMfs2 (T1-4) | PAO1 harbouring pMfs2 containing 4-transmembrane, Ap^r^ | This study |
| PAO1/pMfs2 (T1) | PAO1 harbouring pMfs2 containing 1-transmembrane, Ap^r^ | This study |
| PAO1/pMfs2 (T10-14) | PAO1 harbouring pMfs2 containing 5-transmembrane, Ap^r^ | This study |
| PAO1∆*amgRS* | PAO1 with *amgRS* deletion | This study |
| PAO1∆*amgRS*/p | PAO1∆*amgRS* harbouring pBBR1MCS-4, Ap^r^ | This study |
| PAO1∆*amgRS*/pMfs1 | PAO1∆*amgRS* harbouring pMfs1, Ap^r^ | This study |
| PAO1∆*amgRS*/pMfs2 | PAO1∆*amgRS* harbouring pMfs2, Ap^r^ | This study |
| PAO1*mexX* | PAO1 with *mexX* inactivation by pKNOCK system, Gm^r^ | This study |
| PAO1*mexX*/p | PAO1*mexX* harbouring pBBR1MCS-4, Ap^r^ | This study |
| PAO1*mexX*/pMfs1 | PAO1*mexX* harbouring pMfs1, Ap^r^ | This study |
| PAO1*mexX*/pMfs2 | PAO1*mexX* harbouring pMfs2, Ap^r^ | This study |
| PAO1*armZ* | PAO1 with *armZ* inactivation by pKNOCK system, Gm^r^ | This study |
| PAO1*armZ*/p | PAO1*armZ* harbouring pBBR1MCS-4, Ap^r^ | This study |
| PAO1*armZ*/pMfs1 | PAO1*armZ* harbouring pMfs1, Ap^r^ | This study |
| PAO1*armZ*/pMfs2 | PAO1*armZ* harbouring pMfs2, Ap^r^ | This study |
| PAO1/pTetA(C) | PAO1 harbouring pTetA(C), Ap^r^ | (Leesukon et al., 2013) |
| **Plasmids** | | |
| pKNOCKGM | Gm^r^ | (Alexeyev M. F., 1999) |
| pBBR1MCS-4 | Ap^r^ | (Kovach et al., 1995) |
| pUC18GM | Ap^r^, Gm^r^ | (Somprasong et al., 2012) |
| pCM157 | Tc^r^ | (Marx and Lidstrom, 2002) |
| pMfs1 | pBBR1MCS-4 containing *PA1262* full-length gene, Ap^r^ | This study |
| pMfs2 | pBBR1MCS-4 containing *PA1282* full-length gene, Ap^r^ | This study |
| pMfs1 (T1-10) | pMfs1 with transmembrane 1 to 10, Ap^r^ | This study |
| pMfs1 (T1-6) | pMfs1 with transmembrane 1 to 6, Ap^r^ | This study |
| pMfs1 (T1-3) | pMfs1 with transmembrane 1 to 3, Ap^r^ | This study |
| pMfs1 (T1-2) | pMfs1 with transmembrane 1 to 2, Ap^r^ | This study |
| pMfs1 (T1) | pMfs1 with transmembrane 1, Ap^r^ | This study |
| pMfs1 (T4-14) | pMfs1 with transmembrane 4 to 14, Ap^r^ | This study |
| pMfs2 (T1-8) | pMfs2 with transmembrane 1 to 8, Ap^r^ | This study |
| pMfs2 (T1-4) | pMfs2 with transmembrane 1 to 4, Ap^r^ | This study |
| pMfs2 (T1) | pMfs2 with transmembrane 1, Ap^r^ | This study |
| pMfs2 (T10-14) | pMfs2 with transmembrane 10 to 14, Ap^r^ | This study |
| **Primers (5’→3’)** | | |
| BT5958 | GCACAAGGACTCCGCCCATG |  |
| BT5959 | GCGCCTCAGTTGCCCGGCGA |  |
| BT5960 | GCGACGATCAGTTCCAGTTC |  |
| BT5961 | CGCCACAGAGGTTATCCCATG |  |
| BT5962 | TGGTCGAACTTACCGAGTGT |  |
| BT5963 | ACGATCCAGAGTTTCTCCGC |  |
| BT6022 | GGAAATGCCGACCCGCTGC |  |
| BT6023 | AGCAGCAGCCAGACCGAACC |  |
| BT6024 | CCACCTGCCTCTTCTACCTG |  |
| BT6025 | GTCGTAGTGCTCGCGGATAT |  |
| BT6026 | GATCGCCACCCTCTACGAAG |  |
| BT6027 | TTCATGTTGCGCTACTTCCG |  |
| BT6028 | GGTGATGCCCATTTCCTTCG |  |
| BT6029 | GAAGACATGGCGGACGATC |  |
| BT6223 | GAGTACACCGAAGCGCAGAC |  |
| BT6224 | GGCTGGGAGAAGTTCACGTA |  |
| BT6304 | GGTAGCCCAGGACCAGAATG |  |
| BT6305 | GAACAACCAGGAACCCGAAC |  |
| BT6417 | GAACAAGATCGAGGTCTGCC |  |
| BT6418 | TCGCACTTGAGGTAGAGGATC |  |
| BT7158 | GACAAACTGGAAAAGCGCCT |  |
| BT7159 | CCGTCTCGACCTGCTGTAG |  |
| BT7164 | AGCATCACCCTGAAACTGCT |  |
| BT7165 | GACAGCTCTTCGACGGTTTG |  |
| BT7162 | GATGCTCGACATCCTGTTGAT |  |
| BT7163 | GCGATCTTGTCCATCTCGTT |  |
| BT6423 | GTGCCGATCATCATGCTCAC |  |
| BT6424 | CTCGTCGCCCTTCTTCAGTT |  |
| BT6526 | AGTGAAATGGCCGAGGTGTA |  |
| BT6527 | GATGAAGGCGAGGAACTGGT |  |
| BT7317 | GGAGAGCTCTTCGGCGATAC |  |
| BT7318 | AAGACCGCGGTGCTCGAGGGAAA |  |
| Mfs1 T1-6 | TCATACCCAGCGGCGGCTGCAA |  |
| Mfs1 T1-2 | TCAGTAGTTGTCGCCAAGCCGGCC |  |
| Mfs2 T1-8 | TTACAGGTACAGGCAGAGCATG |  |
| Mfs1 T4-14 | GCACAAGGAGCGTCGCCATGTTCGCCGTCGCCTCGTT |  |
| BT2781 | GCCCGCACAAGCGGTGGAG |  |
| BT2782 | ACGTCATCCCCACCTTCCT |  |
| M13F | GTAAAACGACGGCCAGT |  |
| M13R | GGAAACAGCTATGACCATG |  |
| BT87 | CACTTAACGGCTGACATGG |  |
| BT543 | TGACGCGTCCTCGGTAC |  |

Abbreviations: Km^r^, kanamycin resistance; Ap^r^, ampicillin resistance; Gm^r^, gentamicin resistance; Tc^r^, tetracycline resistance.
Note: Carbenicillin was used for selection of strain harbouring a plasmid vector containing ampicillin resistance cassette (Ap^r^) (Choi et al., 2006).

**Table S2.** BLASTP analysis of MFS among *P. aeruginosa*, *S. maltophilia*, *S.* Typhi (for SmvA and TetA) and *S. aureus* (for NorA and QacA). Data presented are in percent identity.

| **% identity** | **Mfs1** | **Mfs2** | **MfsA** | **SmvA** | **NorA** | **QacA** | **TetA** |
| --- | --- | --- | --- | --- | --- | --- | --- |
| **Mfs1** | 100 | 22.5 | 18.6 | 21.4 | 16.5 | 16.0 | - |
| **Mfs2** | 22.5 | 100 | - | 37.6 | 16.8 | 19.8 | - |
| **MfsA** | 18.6 | - | 100 | 14.8 | - | - | - |
| **SmvA** | 21.4 | 37.6 | 14.8 | 100 | 16.0 | 18.6 | - |
| **NorA** | 16.5 | 16.8 | - | 16.0 | 100 | 17.5 | 20.0 |
| **QacA** | 16.0 | 19.8 | - | 18.6 | 17.5 | 100 | - |
| **TetA** | - | - | - | - | 20.0 | - | 100 |

**Table S3.** MIC values (µg/mL) against PAO1 wild-type of aminoglycosides (gentamicin (CN) and tobramycin (TOB) and quinolones (ciprofloxacin (CIP) and levofloxacin (LEV) in the presence and absence of NaOCl. Data shown are the modes of four independent experiments.

| NaOCl | CN  (µg/mL) | TOB (µg/mL) | CIP (µg/mL) | LEV (µg/mL) |
| --- | --- | --- | --- | --- |
| 0.00% | 1 | 0.5 | 0.125 | 0.5 |
| 0.02% | 1 | 0.5 | 0.125 | 0.5 |


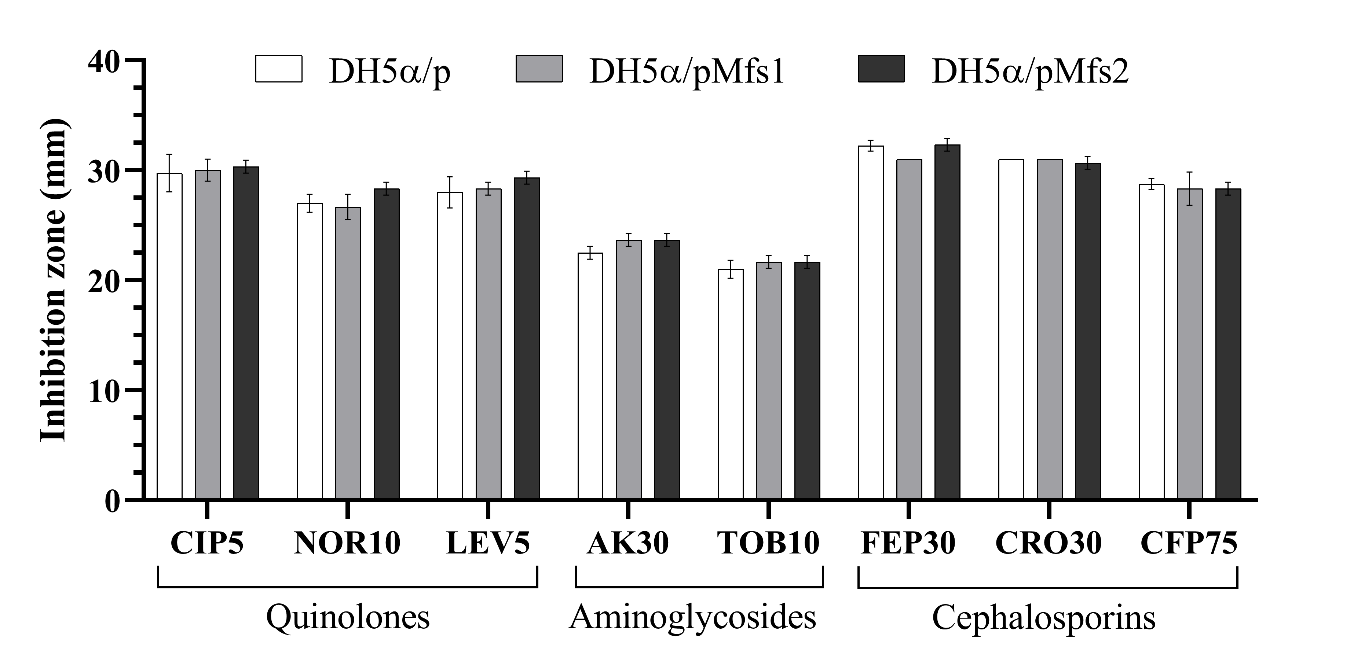


**Figure S1.** Antibiotic susceptibility against the quinolones, ciprofloxacin (CIP 5 µg), norfloxacin (NOR 10 µg), levofloxacin (LEV 5 µg); aminoglycosides, amikacin (AK 30 µg), tobramycin (TOB 10 µg); and cephalosporins, cefepime (FEP 30 µg), ceftriaxone (CRO 30 µg), cefoperazone (CFP 75 µg) for DH5α/p, DH5α/pMfs1 and DH5α/pMfs2. Data presented are means ± standard deviations of three biological replicates. Asterisk (*) indicates significant difference relative to DH5α/p (p-value < 0.05).

**
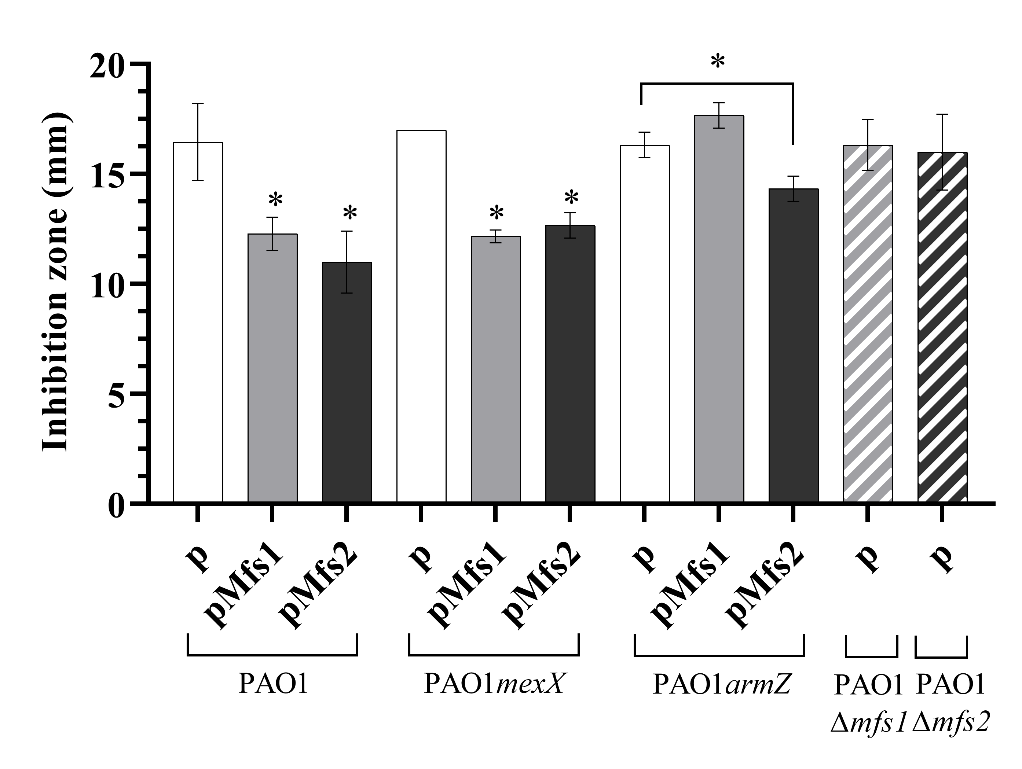
**

**Figure S2.** Paraquat susceptibility profiles of pMfs1 and pMfs2 in PAO1, PAO1*mexX,* PAO1*armZ* and of the absence of Mfs1 and Mfs2 in PAO1∆*mfs1* and PAO1∆*mfs2*. Data presented are means ± standard deviations of three biological replicates. Asterisk (*) indicates significant difference relative to PAO1/p (*p-value* < 0.05).


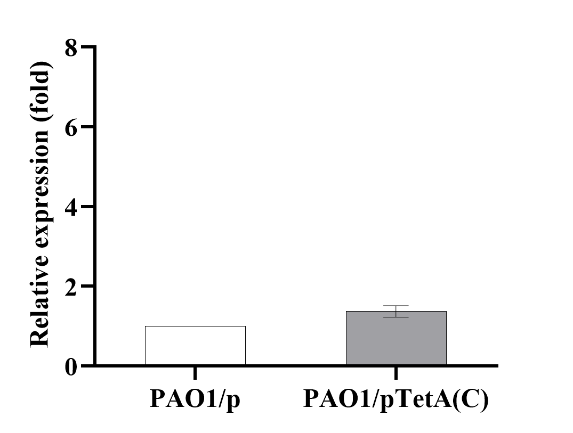

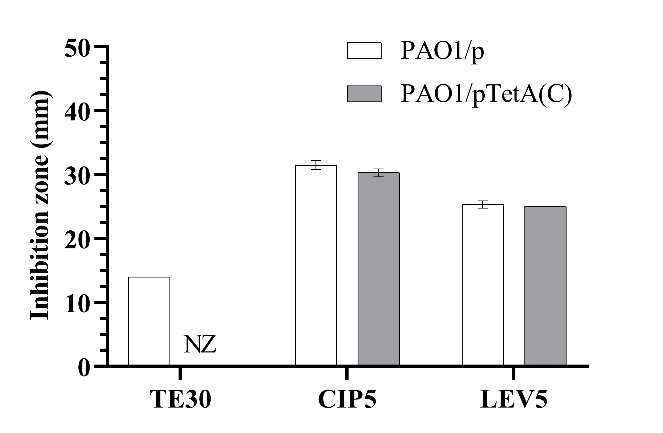


**B**

**A**

**Figure S3.** The antibiotic susceptibility and *mexX* expression levels in PAO1 over-producing TetA(C). In (A), disc diffusion assays were performed to determine the susceptibility of PAO1 harbouring pTetA(C) [PAO1/pTetA(C)] and PAO1 harbouring pBBR1MCS vector control (PAO1/p) toward tetracycline (TE 30 µg), ciprofloxacin (CIP 5 µg), and levofloxacin (LEV 5 µg). NZ, no zone. In (B) the expression of *mexX* was assayed using real-time RT-PCR. Total RNA samples were extracted from exponential-phase cultures of PAO1/p and PAO1/pTetA(C). Real-time PCR of cDNA products were performed using primers specific for *mexX* (BT6223 and BT6224) (Table S1). The *16S rRNA* gene (BT2781 and BT2782) was used a normalising gene expression control. Relative *mexX* expression was defined as the fold change in *mexX* expression in PAO1/pTetA(C) versus PAO1/p, which was set to 1. The data shown were means ± SD from three independent experiments. The change in *mexX* expression seen was not statistically significant.


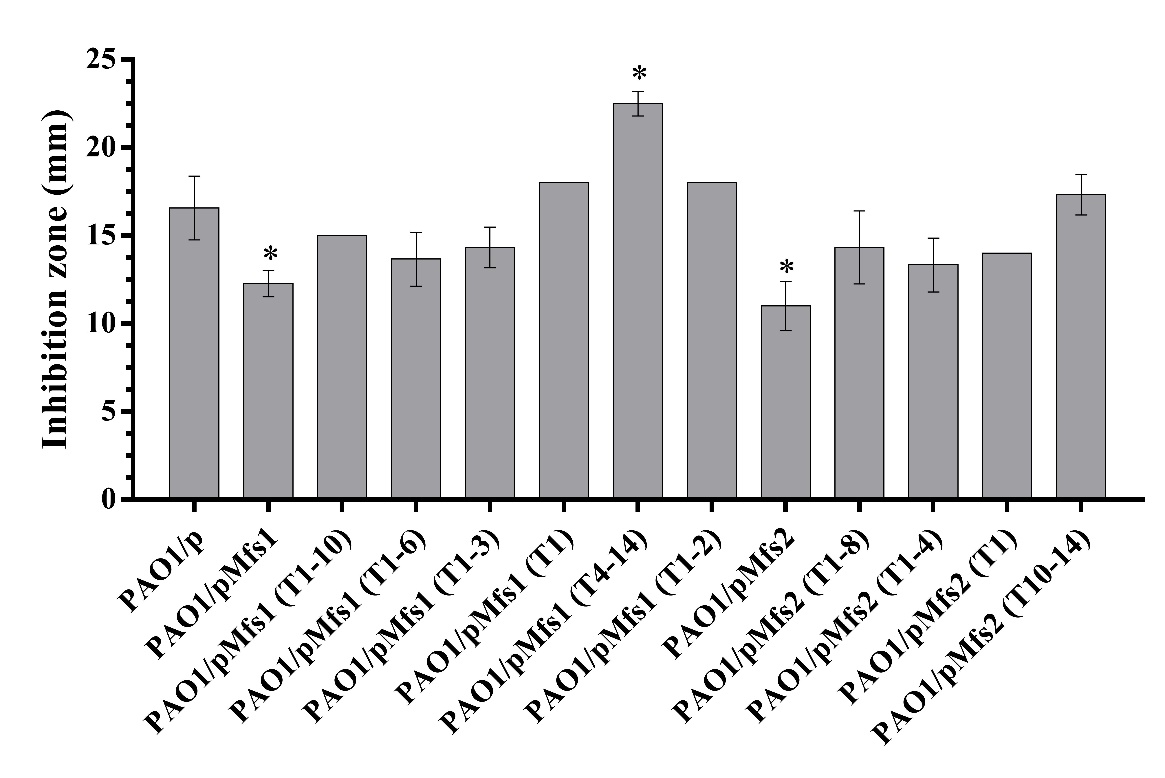


**Figure S4.** Paraquat susceptibility profiles of PAO1 carrying pMfs1 or pMfs2 or truncated versions having various numbers of transmembrane helices. Data presented are means ± SD of three biological replicates. Asterisk (*) indicates significant difference relative to PAO1/p (*p-value* <0.05).

**References**

Alexeyev M. F. (1999). The pKNOCK series of broad-host-range mobilizable suicide vectors for gene knockout and targeted DNA insertion into the chromosome of Gram-negative bacteria. *Biotechniques* 26, 824–828.

Choi, K. H., Kumar, A., and Schweizer, H. P. (2006). A 10-min method for preparation of highly electrocompetent *Pseudomonas aeruginosa* cells: Application for DNA fragment transfer between chromosomes and plasmid transformation. *J. Microbiol. Methods* 64, 391–397. doi:10.1016/j.mimet.2005.06.001.

Kovach, M. E., Elzer, P. H., Hill, D. S., Robertson, G. T., Farris, M. A., Roop, R. M., et al. (1995). Four new derivatives of the broad-host-range cloning vector pBBR1MCS, carrying different antibiotic-resistance cassettes. *Gene* 166, 175–176.

Leesukon, P., Wirathorn, W., Chuchue, T., Charoenlap, N., and Mongkolsuk, S. (2013). The selectable antibiotic marker, tetA(C), increases *Pseudomonas aeruginosa* susceptibility to the herbicide/superoxide generator, paraquat. *Arch. Microbiol.* 195, 671–674. doi:10.1007/s00203-013-0913-7.

Marx, C. J., and Lidstrom, M. E. (2002). Broad-host-range *cre-lox* system for antibiotic narker recycling in Gram-negative bacteria. *Biotechniques* 33, 1062–1067.

Somprasong, N., Jittawuttipoka, T., Duang-Nkern, J., Romsang, A., Chaiyen, P., Schweizer, H. P., et al. (2012). *Pseudomonas aeruginosa* thiol peroxidase protects against hydrogen peroxide toxicity and displays atypical patterns of gene regulation. *J. Bacteriol.* 194, 3904–3912. doi:10.1128/JB.00347-12.
